# Training in the Dark: Using Target Training for Non-Invasive Application and Validation of Accelerometer Devices for an Endangered Primate (*Nycticebus bengalensis*)

**DOI:** 10.3390/ani12040411

**Published:** 2022-02-09

**Authors:** K. Anne-Isola Nekaris, Marco Campera, Marianna Chimienti, Carly Murray, Michela Balestri, Zak Showell

**Affiliations:** 1 Nocturnal Primate Research Group, Oxford Brookes University, Oxford OX3 0BP, UK; mcampera@brookes.ac.uk (M.C.); mbalestri@brookes.ac.uk (M.B.); 2 Centre d’Etudes Biologiques de Chizé, 79360 Chizé, France; mariannachimienti@gmail.com; 3 Shaldon Wildlife Trust, Shaldon TQ14 0HP, UK; carly@shaldonwildlifetrust.org.uk (C.M.); zak@shaldonwildlifetrust.org.uk (Z.S.)

**Keywords:** animal training plan, positive reinforcement, Strepsirrhini, animal welfare, bio-logger, random forest

## Abstract

**Simple Summary:**

Recent advances in technology allow for the study of animal behaviours through indirect observations. This facilitates research on cryptic animals for which direct observations may miss a considerable portion of their activity. The validity of accelerometers in obtaining accurate animal behaviours, however, needs to be tested before collecting data in the wild. Modern zoos offer excellent opportunities for researchers to test field techniques in a safe setting. Here, we describe a non-invasive training program to attach an accelerometer to an individual Bengal slow loris at the Shaldon Wildlife Trust. This training took 39 15-min sessions and allowed for the attachment of the accelerometer for validation with reduced stress for the animal. We also collected videos to associate to accelerometer data to estimate the accuracy of accelerometers in identifying the behaviours of Bengal slow loris. The accuracy was above 80% with some of the behaviours that were clearly identified (e.g., resting: 99.8%), while others were more difficult to discern (e.g., suspensory walk, a locomotion behaviour, was discerned only 60.3% of times from other behaviours). The non-invasive training and accelerometer validation can be used on similar species before using accelerometers in the wild.

**Abstract:**

Accelerometers offer unique opportunities to study the behaviour of cryptic animals but require validation to show their accuracy in identifying behaviours. This validation is often undertaken in captivity before use in the wild. While zoos provide important opportunities for trial field techniques, they must consider the welfare and health of the individuals in their care and researchers must opt for the least invasive techniques. We used positive reinforcement training to attach and detach a collar with an accelerometer to an individual Bengal slow loris (*Nycticebus bengalensis*) at the Shaldon Wildlife Trust, U.K. This allowed us to collect accelerometer data at different periods between January–June 2020 and January–February 2021, totalling 42 h of data with corresponding video for validation. Of these data, we selected 54 min where ten behaviours were present and ran a random forest model. We needed 39 15-min sessions to train the animal to wear/remove the collar. The accelerometer data had an accuracy of 80.7 ± SD 9.9% in predicting the behaviours, with 99.8% accuracy in predicting resting, and a lower accuracy (but still >75% for all of them apart from suspensory walk) for the different types of locomotion and feeding behaviours. This training and validation technique can be used in similar species and shows the importance of working with zoos for in situ conservation (e.g., validation of field techniques).

## 1. Introduction

Remote measurement of animal behaviours has now been made possible through the availability of bio-logging devices such as accelerometers. The reduction in the size of microprocessors and increase in memory storage and battery life means that these devices can now be used on smaller species including arboreal species living in often inaccessible habitats [1]. For taxa that are cryptic, nocturnal, or elusive, traditional behavioural approaches may miss a considerable portion of their activity. Thus, data from accelerometers can reduce observer bias, quantify fine scale movements, and provide detailed data on the energetics of species [2]. At the same time, equipping cryptic taxa with accelerometers can prove challenging, involve multiple recaptures, and ensuring the longevity of devices in often harsh climates.

For these reasons, most accelerometer studies have been conducted on captive, domestic, or aquatic taxa. For arboreal species, the difficulties of catching and attaching loggers may be confounded by trial and error, where the loggers or collar styles fail [3,4]. Retrieving the loggers can place further constraints, and thus may result in low sample sizes in the wild, where the expense and risk of applying loggers must be considered [5]. Furthermore, for species that are difficult to observe continuously, validation in captivity is necessary to analyse the data collected by the loggers [6]. Finally, catching animals and putting a potentially hazardous device on them, a process that may require an equally risky anaesthesia, needs to be considered, especially for species that are globally threatened [4].

One of the key objectives of modern zoos is to aid in the in situ conservation of threatened species. Although this is frequently conducted through public education or the financial support of conservation projects, the potential to trial field techniques in captive animals occurs less often. Zoos, of course, must consider the welfare and health of the individuals in their care, with unnecessary animal handling practices being used as little as possible, and often only during the health checks of animals. At the same time, trialling field techniques such as the application of radio collars or accelerometers can be carried out in a safe setting with a veterinarian on hand. Scientists can then be equipped with the training to carry out such procedures on wild populations of endangered taxa. Furthermore, validation of accelerometers in captivity provides essential baseline data, allowing researchers to more quickly understand data collected in the wild [7].

Positive reinforcement training (PRT) is a form of operant conditioning learning increasingly used by zoos, which involves rewarding animals to elicit specific behaviours [8]. By associating behaviours important for veterinary, husbandry, or scientific procedures with a positive experience (i.e., rewards), the animal can gain motivation to engage in the behaviour [9]. PRT may start with food rewards, but by linking these rewards with an alternative action or associated words, the behaviour can be stimulated by such actions alone. Target training is an increasingly commonly used PRT strategy, whereby an animal is trained to touch or be touched by an object such as a dowel or plastic target stick [10]. These training sessions, for example, are meant to help with the delivery of health checks and reduce the incidence of undesirable behaviours. In addition to the training goal, target training can be an important additional element to enrichment and to increasing the relationship between an animal and a keeper (e.g., by reducing potential risks of bites or scratches for keepers) [11].

Slow lorises (*Nycticebus* spp.) are globally threatened nocturnal primates, nine species of which are found in Southeast Asia, but which are scarce in zoos due to poor neonate survivorship [12]. In the wild, they are frequently found in dense tangles and bamboo thickets, meaning that they are out of view of the researchers for about 30% of the time [13,14]. Their locomotion is evolutionarily unique amongst primates in that they do not leap, and along with their unique slow energetics, their activity patterns and locomotion have long been of interest to researchers [15]. Most captive studies, however, usually record locomotion and energetics on a single substrate that is vastly different to their complex habitat use in the wild [16]. Wild slow lorises readily wear radio collars and tend not to remove them [17,18]. Activity score accelerometers have been successfully applied to wild Javan slow lorises (*N. javanicus*). These accelerometers yield less specific information on activity but have been useful in sleep research [5]. Three-axis accelerometers, however, can provide more information on these cryptic species, for example, lorises are out of sight of direct observation around 30–40% of the time [14], meaning that we are missing a large proportion of behaviours.

The Shaldon Wildlife Trust is the smallest zoo in the U.K. and, in the last two decades, has provided care for wild-born Bengal slow lorises (*N. bengalensis*) confiscated from the illegal wildlife trade. As the closest relative to Javan slow lorises, which do not occur in captivity and have a similar body size, the zoo management agreed to collaborate in undertaking a 3-axis accelerometer study with two objectives. First, we investigated whether target training was an option to train a slow loris to wear a collar fitted with an accelerometer. Second, we sought to use data gained from the accelerometer to validate behavioural data of *Nycticebus* to provide a baseline to apply to the wild. We discuss the results in relation to the welfare and ecological research of cryptic species and how zoological collections and researchers can work together to achieve these ends.

## 2. Materials and Methods

### 2.1. Data Collection

We conducted the research at the Shaldon Wildlife Trust, Devon, United Kingdom, in the zoo’s nocturnal house. Animals are kept on a reverse light cycle, with twelve hours of nocturnal illumination provided by a combination of red and blue LED lights from 600 h to 1800 h. The Bengal slow loris enclosure is approximately 205 cm long, 61 cm wide and 91 cm high, and is enriched with a mix of fixed and dynamic branches and logs of various sizes providing platforms for continuous movement. The enclosure was thoroughly checked for potential hazards prior to beginning the experiment. Food is provided on wooden platforms, with gum provided in logs with pre-drilled holes. The temperature of the enclosure is approximately 24 °C. The subject of the study was a single adult female Bengal slow loris (ZIMS ID 1190; call name Tina), weighing ~1500 g at the time of study. Tina was wild caught for the illegal pet trade. After rescue, she was imported from the Kadoori Gardens in Hong Kong and arrived at the Shaldon Wildlife Trust on 19 December 2016. Despite being rescued from the pet trade, Tina maintains a positive relationship with the zookeepers and does not display any abnormal behaviour towards them such as aggression or fear. When she initially arrived at the Shaldon Wildlife Trust, she was quiet and shy, but following the death of her enclosure mate, her confidence grew, and she was more willing to approach zookeepers and actively participate in training sessions.

To gain confidence and trust with keepers and to facilitate routine care, training with Tina began on 19 July 2017. She was introduced to several elements: a tong feeding stick, target stick, weighing scale, and crate training. The first stage was to attract the animal to retrieve an insect from a tong feeding stick accompanied with a bridge—a sound that the animal can associate with a reward [10]. Although the standard Shaldon Wildlife Trust protocol is to use a training clicker as a bridge, keepers found this too loud for slow lorises and the term “good” was introduced as the bridge. Training sessions lasted approximately 15 min. The next approximation was to get the slow loris to touch a target stick with her mouth or hand, first by having an insect on the stick, and then by touching the target stick on its own. She was thus familiar with these training devices when we used six stages to train her to wear a snap-on safety cat collar on 29 October 2019 (Table 1 and results). We completed this training on 7 January 2020. We chose a snap-on safety cat collar for the training as it was easy to take on and off quickly and had a quick release mechanism if for any reason it became stuck on the cage furniture.

After training, the cat collar was equipped with a three-axis accelerometer (Technosmart, Axy 5 S) with dimensions of 22 mm × 13 mm × 10 mm, weighing 4.5 g (thus well below the 5% of body weight threshold suggested when collaring animals). The accelerometer was set to collect data at a frequency of 25 Hz. We collected data between January–June 2020 and January–February 2021, totalling 42 h of videos, from which we collected the data for validation. Two separate night vision video cameras were positioned to ensure the best possible view of the behaviours of the animal, but the animal remained still out of sight for most of the time or the behaviours were not clear, so we managed to associate 54 min of video to the accelerometer data. We used the same ethogram we used for wild Javan slow lorises and recorded as many behaviours as possible [14]. We were able to obtain extensive details on ten behaviours (Table 2).

### 2.2. Data Analysis

From the raw data collected by the accelerometer, we calculated the static and dynamic acceleration, amplitude (i.e., standard deviation of the dynamic acceleration), ODBA (overall dynamic body acceleration) and pitch by using the package “plotrix” over a smoothing factor of 2 s to reduce noise [19]. The full dataset with associated behaviours used for the analysis contained 81,051 datapoints. We first created a new dataset with an even number of datapoints per each category (i.e., we reduced, using a random selection, the number of datapoints for each behaviour to match the number of datapoints of the less frequent behaviour). This was to avoid unbalanced categories that might have biased the classification. We created two random datasets: one training and one validation set containing 70% and 30% of the data, respectively. We performed the random forest on the training set with the function “randomForest” from the package “randomForest” [20]. The behaviours were set as the response variable and the variables obtained from the accelerometers were the predictors in the random forest classifier. We calculated the optimal mtry (i.e., number of features used in the construction of each tree) via the function “tuneRF”. We obtained the importance of each variable in the random forest classifier via the function “importance” and plotted with the function “varImpPlot”. We then used the random forest to predict the validation set via the function “predict” and reported the accuracy of predictions. A detailed description of the use of random forest models for validation can be found in Dickinson et al. [21]. The analyses were performed via R v 4.1.0.

## 3. Results

### 3.1. Training for Application of the Device

**Figure 1:**
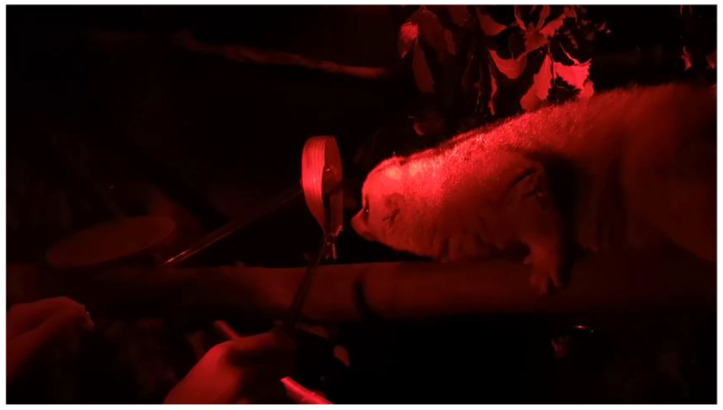
Tina the Bengal slow loris being presented with the bamboo leaf collar with tongs during the initial stage of the training.

Stages of training are summarised in Table 1. In order to train Tina to become accustomed to having the collar placed over her head and around her neck, we presented the target stick as a focus on the other side of the collar. Before introducing the cat collar, we initially introduced a larger bamboo leaf in the shape of a collar that was larger than the diameter of her head, so she could move in and out freely. We held the target stick in the centre of the collar to encourage Tina to move her head towards it; once she touched the target with her nose or was close, we gently held the collar over her head for a few seconds (Figure 1).

At this point, we began to touch Tina’s neck with the cat collar. This stage took several attempts as Tina needed to adjust to the new collar. Any time she regressed, we moved a step back and rewarded her for just touching the target stick to keep her engaged. After 12 sessions, she began to push her head through the collar to touch the target stick.

**Figure 2:**
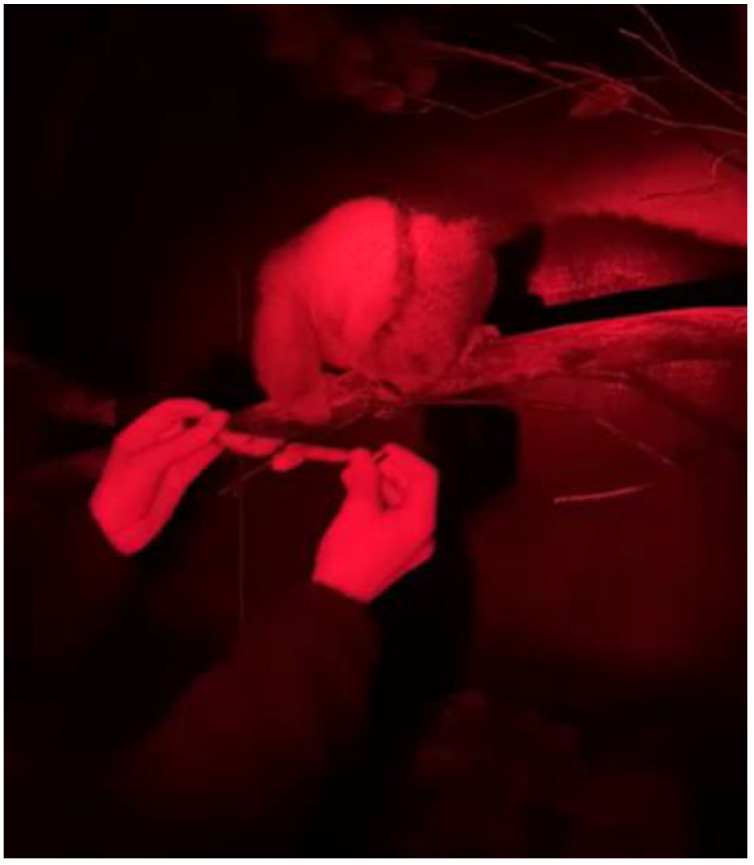
Tina the Bengal slow loris at the final stage of the training when we were able to safely cat collar and equip her with the accelerometer device with our hands.

This allowed Tina to choose to touch the collar herself by moving her head through the loop to touch the target stick on the other side. The target stick was slowly moved further out of the loop of the collar away from Tina on each session so that she had to reach further through the collar. We then progressed to placing the collar directly over Tina’s head using the tongs once she had pushed her head through to touch the target stick. The collar was adjusted so that it was relatively loose to allow us to wiggle the collar from side to side to fit over her neck. After only two sessions, we could place the collar fully over her head (Figure 2).

Slow lorises can bite and are also venomous, so we deemed it prudent also to remove the collar with tongs. To achieve this, we used the words “Tina, touch” as a prompt that something was about to happen, and then to introduce a new command. We used the tongs to manipulate the collar whilst it was on her head, after which we said “touch”, and gently moved the collar around on her neck. She allowed this in the first session, but to reinforce the behaviour, we added three additional sessions. After this, we added the accelerometer to allow Tina to acclimatise to the smell and weight. As reinforcement, we allowed her to eat her preferred insects whilst wearing the collar for a few minutes at a time.

For ease of fitting and removing the collar, we next trained Tina to allow this to be undertaken by hand. This training also distinguished the collar work from general feeding, when keepers always wear gloves. Saying the touch command, we started to replace the tongs with the keeper’s left hand, held up flat vertically facing Tina’s face with the palm forward. We followed this by gently placing the right hand over the back of her neck and moving the collar. She was wary at first, so we introduced this keeper touch command without the collar on intermittent days to the collar training. This involved using the target command to start the session to reinforce her, followed by using the flat hand command, saying “Tina, touch”, and touching the back of her neck with the right hand, applying very gentle pressure each time. She was rewarded with the term “good” if she sat still during this process. It took her 12 sessions to acclimatise to being touched, with each session being slightly longer and touching her neck more to mimic wearing the collar. To start her becoming used to wearing the actual collar, we held the collar flat in front of her and gently touching underneath her neck and using the “touch” command. After two sessions, she allowed us to mimic clipping the collar around her neck. With the success of the training, Tina wore the collar during her active period. Despite biting at the accelerometer three times after it was initially placed on her, with each bout of biting decreasing in severity and longevity, she quickly grew used to it and allowed us to remove it during her inactive periods on a regular basis. The collar was removed only when the animal was conscious and had approached a zookeeper to minimise the level of disturbance.

### 3.2. Accelerometer Validation

**Figure 3:**
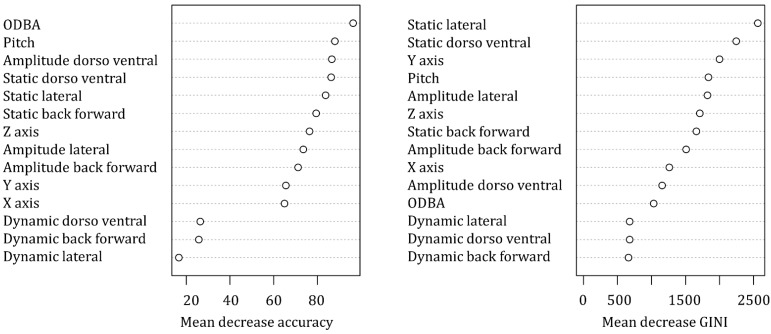
Mean decrease accuracy and mean decrease GINI of the predictor variables included in the random forest classifier.

**Figure 4:**
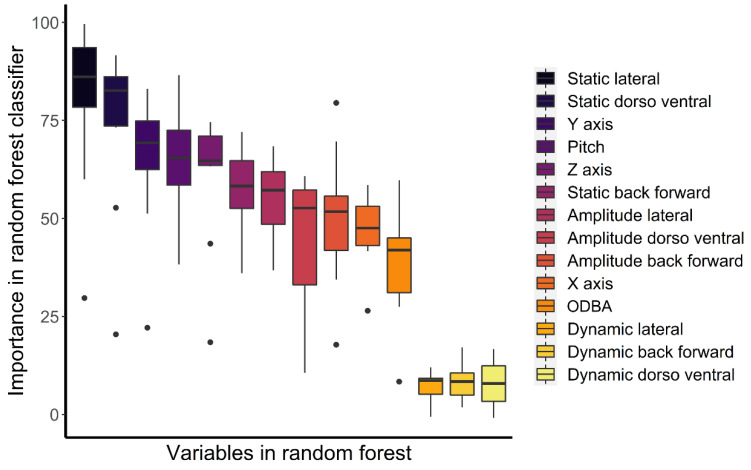
Box plots of the importance as a classifier of the variables included in the random forest. Values are medians, quartiles, and ranges considering the ten behaviours tested. Points are outliers.

The variables associated with the accelerometers had an accuracy of 80.7 ± SD 9.9% in predicting the ten behaviours of the captive Bengal slow loris (Table 3). The highest accuracy was obtained with resting behaviour (99.8%), while the lowest accuracy was obtained for suspensory walk (60.3%), which was mainly confused by the algorithm with suspensory feeding (18.8% of suspensory walk from the validation set was associated with suspensory feeding). ODBA was the most important variable to predict behaviours based on the mean decrease accuracy (i.e., estimate of the loss in prediction performance when a variable is omitted from the training set; Figure 3), while static lateral acceleration was the most important classifier (Figure 4) and had the highest mean decrease GINI (i.e., node impurity, so the higher it is, the more important the variable is to split the data correctly). Furthermore, static lateral acceleration was the most important classifier for seven behaviours out of ten. The only behaviour that was poorly classified by the static lateral acceleration was resting, where the amplitude of the lateral dynamic acceleration was more important. The body pitch was an important predictor for specific behaviours such as resting, bridge, walk, and feeding non-suspensory. Dynamic accelerations had the lowest importance as classifiers.

## 4. Discussion

In just 39 15-min sessions, we were able to train a slow loris to adjust to a novel stimulus—wearing a cat collar with an accelerometer—using target training. Through this non-invasive method, we were additionally able to collect sufficient accelerometer data to validate ten common behaviours of slow lorises. The target training was also a novel form of enrichment for the animal in question and allowed a zoological collection to provide a unique contribution to in situ research without compromising the health or welfare of an animal. We also contribute to the growing body of literature on training in primates, and especially in strepsirrhines [22,23].

Training is becoming increasingly common in zoos to facilitate husbandry and veterinary needs. For example, PRT was used with rhesus macaques (*Macaca mulatta*) in a research facility to manage and improve keeper care time [24]. North American porcupines (*Erethizon dorsatum*) were trained to enter squeeze boxes to receive subcutaneous injections [25]. Shaping training has been used with chimpanzees to improve welfare and reduce stereotypic behaviours [26]. Fuller et al. [22] trained pygmy slow lorises (*N. pygmaeus*) to chew on swabs to collect saliva for a welfare study understanding the impact of different coloured lights on their physiology. Here, we provide a novel example of PRT whereby a slow loris was trained to wear a collar for the purpose of collecting data for ecological research. Previous work has suggested that due to the trust that must be developed between the two, positive interactions between caretakers and primates are likely to develop during PRT [27]. Indeed, not only did the slow loris in question show no negative behavioural effects, but the training itself was a form of enrichment and Tina developed increased trust with her main keeper. For a venomous animal such as a slow loris, which has been known to bite keepers resulting in subsequent hospitalisations [28], increasing a friendly keeper bond should also be seen as positive [29]. As has been observed in other studies, Tina acclimatised rapidly to the training sessions and participated willingly (c.f., [23]). We hope that by sharing these results, more zoos will be willing to collaborate with in situ researchers who might be using novel technologies for the first time. Allowing researchers to perfect these activities in captivity not only saves valuable time in the field but can help to troubleshoot aspects of the technology in a more stable setting where veterinary care is available.

All species of slow lorises are globally threatened. It was thus important for us to know that a loris could wear the logger, which is attached close to their skin, safely. Indeed, Tina showed no adverse health effects or abrasions from the collar. We note that collars have also been used in wild lorises for ten years, and we did not record any adverse health effects or abrasions during our regular health checks [18]. We also could learn how to attach the logger to the collar and how secure it needed to be held to the neck. This is important, as in the wild, the catchability of species varies, and losing a valuable chance to attach a collar or a logger through lack of practice can result not only in loss of data, but in undue stress to the animal. For example, Allan et al. [30] were able to catch and collar 32 bobuck, which they could follow for 149 nights. They reported no loss of collars or of animals due to wearing them. In the case of colugos, however, of the six animals tagged with a glue-based device, only five retained the device on initial collaring, with the others losing it within 1–4 weeks [31]. Testing the efficiency of accelerometers before use in the wild is also important as there might be problems with the devices. For example, Reinhardt et al. [5] pointed out that only seven of the twelve retrieved accelerometers had complete data stored, while the others had skewed data. Campera et al. [4] also noted that the average battery time of accelerometers could be reduced (from one year to 1–4 months) in extreme field conditions such as heavy rains.

The three-axis accelerometers were extremely accurate in discerning between resting and active behaviours (99.8% accuracy). This matches the study on the wild arboreal primates Southern woolly lemurs *Avahi meridionalis* (98.6–99.4% accuracy) and Fleurette’s sportive lemurs *Lepilemur fleuretae* (98.2–99.3 accuracy) [5]. The accelerometer data were, however, less accurate in discerning the different feeding (75.0–85.6% accuracy) and locomotion postures (60.3–85.9% accuracy). This was expected as arboreal primates have very complex postures that often change quickly and have similar kinematic features [32]. Fehlmann et al. [33] managed to obtain higher accuracies for locomotion types and feeding of the terrestrial primate chacma baboons *Papio ursinus* (~88% accuracy), but only discerned between foraging, running, and walking as terrestrial primates have less complex locomotion and feeding postures than arboreal primates. Higher accuracies in discerning feeding and locomotion postures can be found on some terrestrial mammals such as Bovidae Alpine ibex *Capra ibex* and domestic pygmy goat *Capra aegagrus hircus* (~98% accuracy) [21]. In apex predators, discerning type of locomotion and feeding is more complex. For example, in captive pumas *Puma concolor*, Wang et al. [34] were only able to discern between resting (96.8% accuracy), low and high frequency movements (92.0–93.8% accuracy) but did not discern between the different movements and had a low accuracy for feeding (63.7%). In captive dingos *Canis dingo*, Tatler et al. [35] managed to discern 14 behaviours with an overall accuracy of 87% and suggested that a random forest model had better performances than other classification methods. The importance of the variables included in the random model also change between species, thus suggesting the importance of species-specific validation. It is evident that there is still a lot to work to conduct to validate the use of accelerometers for some species and on the variation of behaviours on a large sample size to increase accuracy, but studies such as ours can help researchers working on similar species.

## 5. Conclusions

In this paper we first described a training programme for non-invasive application of accelerometer devices to an individual Bengal slow loris, and then showed the results of the validation to be able to then use the accelerometers on wild slow lorises. Finding non-invasive techniques to trial field techniques in captive animals is increasingly important given the increased concern over animal welfare and safety. We provide an example of positive reinforcement training used for accelerometer validation that can be easily applied in other species. We also provide an example of random forest models used to validate accelerometer data, with high accuracy in discerning between active and inactive behaviours and a lower accuracy (although comparable to other species) in discerning between the different locomotory and feeding postures in the arboreal Bengal slow loris. We believe that the validation described in this paper provides evidence that the main behaviours of wild lorises can be obtained via 3-axis accelerometers. Still, we suggest that researchers use additional validation techniques in the field (e.g., videos and behavioural observations) to further support the findings as some behaviours (e.g., locomotion) might change between in situ and ex situ conditions. Researchers might also consider alternatives to accelerometers and other loggers where ethical issues may arise [36]. We highlight the importance of species-specific validation of accelerometer data that should be conducted in a non-invasive environment to reduce the stress of captive animals.

## Figures and Tables

**Table 1:** Description and duration of the seven stages of positive reinforcement and target training of a Bengal slow loris to wear a collar with an attached accelerometer from 29 October 2019 to 7 January 2020.

Stage	No. Training Days	Description
1	---	As part of regular daily management, feed insects from tongs with the bridge word “good”.
2	5	Presenting target stick with insect reward.
3	4	Accepting placement of large bamboo collar over the head from the target stick.
4	12	Learning to push head through quick release cat collar from the target stick.
5	2	Keeper places cat collar over loris’ head with the target stick.
6	3	Introducing the touch command to remove the collar with accelerometer with the tongs.
7	12	Keeper touch with no collar on to become used to being touched by hands.
8	2	Clipping the collar on and off with the hands.

**Table 2:** Slow loris behavioural ethogram used in this study for video data collection and accelerometer validation.

Behaviour	Locomotion/Posture	Description
Alert	Sit/stand	Remain stationary such as in “rest” but active scanning of environment.
Explore		Movement associated with looking for food (often includes visual and olfactory searching) or exploring the habitat.
	Bridge	Exploring while climbing from one support to the next, (trunk or branches of same or different trees), stretching over a gap of more than 15 cm.
	Climb down	Exploring while moving downwards on +/−45° to 90° support.
	Climb horizontally	Exploring while moving horizontally through +/−90° or +/−45° support.
	Climb up	Exploring while moving upwards on +/−45° to 90° support.
	Suspensory walk	Exploring while hanging on +/−0° to 45° support.
	Walk	Exploring while walking quadrupedally on +/−0° to 45° support.
Feeding		Actual consumption of a food item.
	Non-suspensory	Feeding in a stationary position (e.g., sit or stand).
	Suspensory	Feeding in a suspensory position while hanging from a branch.
Resting	Sit/stand	Remain stationary, often with body hunched

**Table 3:** Results of the random forest classification to assess the predictive power of the variables retrieved from a three-axis accelerometer in assessing the behaviours of a captive Bengal slow loris. Prediction accuracy and main confusing behaviour were based on the performance of the random forest model obtained from the training set of data in predicting the behaviours in the validation set. The importance in random forest classifier was based on the training set.

Behaviour	Prediction Accuracy (%)	Main Confusing Behaviour (% error)	Importance in Random Forest Classifier		
			1st Variable	2nd Variable	3rd Variable
Resting	99.8	Suspensory walk (0.2)	Amplitude lateral	Pitch	Static back forward
Bridge	85.9	Suspensory walk (4.3)	Pitch	Static lateral	Static dorso ventral
Suspensory feeding	85.6	Suspensory walk (5.1)	Static lateral	Static dorso ventral	Y axis
Climb down	82.5	Walk (8.7)	Static lateral	Static dorso ventral	Y axis
Climb up	80.7	Walk (9.6)	Static lateral	Static dorso ventral	Y axis
Alert	80.4	Walk (9.3)	Static lateral	Static dorso ventral	Amplitude back forward
Climb horizontally	79.8	Suspensory walk (15.5)	Static lateral	Static dorso ventral	Amplitude back forward
Walk	77.2	Alert (9.6)	Static lateral	Pitch	Static dorso ventral
Feeding non-suspensory	75.0	Suspensory feeding (9.0)	Static lateral	Static dorso ventral	Pitch
Suspensory walk	60.3	Feeding suspension (18.8)	Static lateral	Static dorso ventral	Z axis

## Data Availability

Data can be made available by the corresponding author by request.
